# Different Arms of the Adaptive Immune System Induced by a Combination Vaccine Work in Concert to Provide Enhanced Clearance of Influenza

**DOI:** 10.1371/journal.pone.0115356

**Published:** 2014-12-18

**Authors:** Joanna C. A. Cobbin, Weiguang Zeng, David C. Jackson, Lorena E. Brown

**Affiliations:** Department of Microbiology and Immunology, The University of Melbourne at the Peter Doherty Institute of Infection and Immunity, Parkville, Victoria, Australia; Centers for Disease Control and Prevention, United States of America

## Abstract

Current split influenza virus vaccines that induce strain-specific neutralising antibodies provide some degree of protection against influenza infection but there is a clear need to improve their effectiveness. The constant antigenic drift of influenza viruses means that vaccines are often not an exact match to the circulating strain and so levels of relevant antibodies may not be sufficiently high to afford protection. In the situation where the emergent influenza virus is completely novel, as is the case with pandemic strains, existing vaccines may provide no benefit. In this study we tested the concept of a combination vaccine consisting of sub-optimal doses of split influenza virus vaccine mixed with a cross-protective T-cell inducing lipopeptide containing the TLR2 ligand Pam2Cys. Mice immunised with combination vaccines showed superior levels of lung viral clearance after challenge compared to either split virus or lipopeptide alone, mediated through activation of enhanced humoral and/or additional cellular responses. The mechanism of action of these vaccines was dependent on the route of administration, with intranasal administration being superior to subcutaneous and intramuscular routes, potentially through the induction of memory CD8^+^ T cells in the lungs. This immunisation strategy not only provides a mechanism for minimising the dose of split virus antigen but also, through the induction of cross-protective CD8^+^ T cells, proves a breadth of immunity to provide potential benefit upon encounter with serologically diverse influenza isolates.

## Introduction

The World Health Organization has estimated that seasonal influenza is responsible for about 3–5 million cases of severe illness worldwide and about 250,000–500,000 deaths annually. Furthermore, influenza pandemics, which can occur when antigenically novel animal influenza viruses undergo genetic changes that allow them to spread within the human population, pose an additional threat with significant morbidity and death tolls ranging from 1 million to more than 50 million when these viruses first emerge. While immunisation is the most cost-effective way to limit the impact of influenza across the community, a very comprehensive analysis of vaccine efficacy data [1], [2], shows that the protection afforded by the currently used trivalent inactivated split-influenza virus (TIV) vaccines is sub-optimal and inconsistent in the young and elderly, and the live-attenuated influenza virus (LAIV) alternative, while highly protective for young children, shows little efficacy in the rest of the population.

There is a recognised need to develop a different style of vaccine that induces robust and durable protection against influenza with the added property of being effective against different IAV subtypes and strains. Achieving broadly crossreactive immunity depends on invoking alternate types of immune effectors to the highly specific neutralizing antibody induced by inactivated vaccines. To this end, much attention has been focused on generating cross-reactive antibody immunity to conserved regions of IAV proteins such as the extracellular domain of M2 [3]–[6] or the HA stalk [7]–[10]. We and others have attempted to harness the cross-reactive properties of influenza-specific T cells, which can recognise epitopes from the conserved internal proteins of the virus [11]–[14]. CD8^+^ T cells have been linked to effective immunity in humans against an emergent pandemic virus, and in the absence of specific antibody, correlate inversely with the duration of viral shedding [15] and with protection against symptomatic infection [16]. Recent clinical trials involving vaccination with the influenza nucleoprotein and matrix protein expressed from a pox-virus vector followed by challenge with infectious virus have recapitulated these effects [17].

We have previously shown that a particularly effective way of inducing memory CD8^+^ T cell responses is by use of an epitope-based lipopeptide vaccine in which the lipid, dipalmitoyl-S-glyceryl-cysteine (Pam2Cys), is covalently attached to influenza-specific CD4^+^ T cell and CD8^+^ T cell epitopes [12], [18], [19]. The Pam2Cys lipid engages TLR-2 on the surface of dendritic cells (DC) leading to efficient DC maturation. The endocytic properties of TLR-2 aid in antigen loading of DC and allow for prolonged presentation of epitopes to T cells for priming. Intranasal (i.n.) immunisation of mice with these lipopeptides elicits a highly potent population of resident memory CD8^+^ T cells in the lungs [18] which can be rapidly recalled upon viral challenge for up to nine months post vaccination and provide a reduction of pulmonary virus load by several logs [12], [18], [19]. The induced CD8^+^ T cells kill peptide-presenting cells in vivo [12], [18], [19] and can prevent death in mice challenged with A/Puerto Rico/8/34 (H1N1) [20], which is highly lethal in this species.

The very nature of the infected cell lysis function of CD8+ T cells means that the host must be infected by the challenge virus for this type of immunity to be invoked. In a similar manner, conserved epitopes recognized by cross-reactive antibodies are often more available on the surface of infected cells rather than on the virus itself so that their effects are mediated on the infected cell by complement mediated lysis or antibody-dependent cellular cytotoxicity [21], [22]. For this reason vaccines designed to only induce such mechanisms may not be as effective in protecting against the initiation of infection by a well-matched seasonal influenza virus as those available at present. An ideal vaccine might therefore invoke the protective properties of highly strain-specific neutralizing antibodies to the key antigenic sites on the viral HA and also cross-reactive immunity to blunt the effects of infection by serologically distinct strains. This study seeks to test the hypothesis that adding a T cell-inducing component to the split influenza virus vaccine will allow these two arms of the adaptive immune response to act in concert, as opposed to diverting the humoral immune system and weakening its impact against the homologous virus. We also explore whether the adjuvant system used to deliver the T cell epitopes can increase the robustness of the co-induced antibody responses. We report on the use of combination vaccines containing split virus at doses that are sub-optimal for complete virus load reduction mixed with the influenza-specific lipopeptide and show these greatly enhance viral clearance from the infected lung.

## Materials and Methods

### Influenza virus

In this study we used the well-characterised Mem-Bel (H3N1) virus [23], a genetic reassortant bearing the NA of A/Bellamy/42 (H1N1) virus and the remaining genes of the A/Memphis/1/71 (H3N2) virus. Virus stocks were propagated in the allantoic cavity of 10-day-old embryonated hen's eggs and three days later harvested and stored at −80°C. Infectious virus was enumerated by plaque formation in Madin-Darby canine kidney (MDCK) cells [24]. The preparation of split virus was prepared at bioCSL Ltd. (Parkville, Victoria, Australia) using a scaled-down method similar to that used in vaccine production. Allantoic fluid was clarified and concentrated by ultrafiltration and inactivated with β-propiolactone (Ferak Berlin GmBH, Berlin, Germany). The virus was disrupted in sodium taurodeoxycholate detergent using proprietary technology (bioCSL Ltd) and the detergent removed via ultra-filtration using a 10,000 MW cut-off membrane in a Miniflow 10 cartridge (Millipore, Billerica, MA, USA). The total virion protein concentration was determined by bicinchoninic acid protein assay (Pierce Chemical Company, Rockford, IL, USA) with HA representing 30% of the total.

### Synthetic lipopeptide

The method of assembling, purifying and characterising lipopeptides has been detailed previously [12], [25]. The influenza specific lipopeptide construct incorporates a peptide representing the CD4^+^ T cell epitope (GALNNRFQIKGVELKS) from the HA2 chain of all H3 viruses [26] and a peptide representing the CD8^+^ T cell epitope (TYQRTRALV) present in the NP protein of PR8 (NP_147-155_) and conserved in all influenza type A viruses [27]. The CD4^+^ and CD8^+^ T cell epitopes were linked via a lysine residue and the Pam2Cys attached to the lysine through two serine residues to form a branched structure.

### Immunisation of mice

Inbred 6–8 week old BALB/c (H-2^d^) mice were maintained in specific pathogen-free conditions in the Animal Facility of the Department of Microbiology and Immunology, University of Melbourne. Mice were immunised once with the indicated dose either by i.n. route with 50 µl of vaccine dispensed onto the nares of mice under light penthrane anaesthesia resulting in deposition in both the upper and lower respiratory tract, by the sub-cutaneous (s.c.) route with 100 µl of vaccine administered at the base of the tail, or by the intramuscular (i.m.) route with 25 µl of vaccine administered to each thigh.

### Viral challenge and lung sample preparation

One month post vaccination, mice were inoculated i.n. with 10^4.5^ plaque-forming units (pfu) of infectious virus under light penthrane anaesthesia. On day five post-infection, mice were killed by cervical dislocation and lungs collected in serum free RPMI 1640 (Gibco, Gaithersburg, MD, USA) supplemented with antibiotics.

### Ethics statement

Mouse experiments were approved by the Biochemistry & Molecular Biology, Dental Science, Medicine and Microbiology & Immunology Animal Ethics Committee of the University of Melbourne and conducted according to the Australian Code of practice for the Care and Use of Animals for Scientific Purposes, 7th edition.

### Lung sample analysis

Single cell suspensions of lung samples were prepared by passing the lungs through a metal sieve. Following centrifugation to pellet the cells, the supernatants were collected and the infectious virus titre determined by plaque formation on MDCK cells monolayers. Influenza-specific CD8^+^ T cells in the cell fraction were enumerated by intracellular cytokine assays (ICS) for interferon γ-secreting CD8^+^ cells specific for the CD8^+^ T cell epitope in the lipopeptide [20]. Cells, at 2×10^7^ cells/ml, were incubated with or without peptide (TYQRTRALV), in the presence of BD GolgiPlug (BD Biosciences, Franklin Lakes, NJ, USA) for 6 hrs at 37°C in 5% CO_2_. Cells were then stained with fluorescein isothiocyanate (FITC)-conjugated rat anti-mouse CD8 antibody (clone 53–6.7; BD Biosciences), then fixed and permeablised with the BD Cytofix/Cytoperm kit according to the manufacturers instructions (BD Biosciences). Following fixation and permeablisation, cells were washed then stained with PE-conjugated rat anti-mouse IFN-γ antibody (clone XGM1.2, BD Biosciences), for 30 min at 4°C. Data were acquired using a BD FACSCalibur flow cytometer (BD Biosciences) and analysed with CellQuest Pro software (BD Biosciences).

### Assay of virus-specific antibody production

Mice were bled via the tail vein on the day prior to challenge and serum samples used to examine virus-specific antibody by enzyme-linked immunosorbent assay (ELISA) or hemagglutination inhibition (HI). The ELISA assay [28] utilised polyvinyl flat-bottom microtitre plates (Dynex Technologies Inc., VA) coated with 5 µg/ml HA equivalents of split virus. The absorbance of the solutions was then determined using a Labsystems Multiscan Multisoft microplate reader (Lab-systems, Helsinki, Finland). The optical density was calculated as the absorbance reading at (405 nm–450 nm). Antibody titres were expressed as the reciprocal of the serum dilution giving an optical density of 0.2, which represents 5-times the background level. The HI assay was performed in a microtitre format with 1% chicken red blood cells by standard methods [29].

### Statistical analysis

Data were analysed using Prism 5 software (GraphPad Software Inc., San Diego, CA, USA). A One-Way ANOVA, with Tukey's Multiple Comparison Test for post analysis, was used to assess statistical significance unless indicated otherwise.

## Results

### Determination of sub-optimal doses of lipopeptide and split virus vaccines

**Figure 1:**
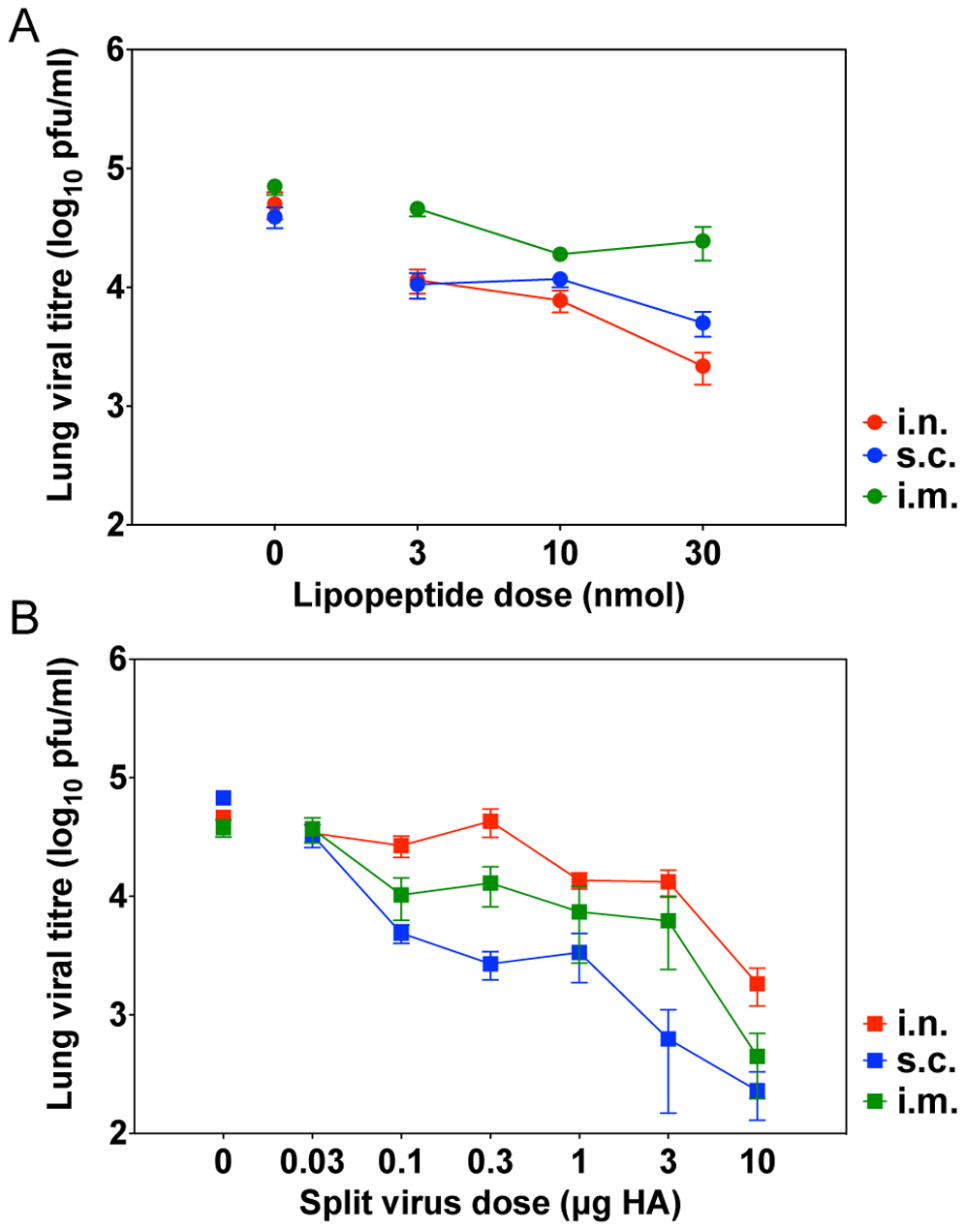
Pulmonary viral clearance after vaccination with lipopeptide or split virus vaccines. Mice were immunised by the i.n. (red), s.c. (blue) or i.m. (green) route with incremental doses of either lipopeptide (A) or split virus (B) on one occasion. One month post-immunisation, mice were challenged by the i.n. route with 10^4.5^ pfu of virus. The lungs were removed 5 days later and pulmonary viral titre determined by plaque formation. Symbols represent the geometric mean for the group (n = 5) and error bars indicate the standard error.

The greatest impact of having a vaccine that could induce or recall memory CD8^+^ T cell responses would be when the antibody levels are insufficient to neutralise the challenge virus. Likewise, the synergistic effect of antibody and T cell responses may mean that sub-optimal lipopeptide doses could also be used. Preliminary dose titrations were therefore undertaken to assess the effect of a single administration of various doses of the two components alone on viral clearance from the lung. Mice were challenged one month after vaccination and lung viral titres assessed 5 days later (Fig. 1). Experiments were performed in mice immunised by either the i.n., s.c. or i.m. routes.

Fig. 1A shows that the route of lipopeptide administration had a significant effect on the efficacy of the lipopeptide (p<0.0001, by two-way ANOVA) with i.n. showing similar levels of viral clearance to s.c. delivery and more effective clearance than i.m. delivery. Lipopeptide doses of 3, 10 and 30 nmol were all effective at providing significant levels of viral clearance (p <0.01) compared to the diluent control, with the exception of the 3 nmol dose administered by the i.m. route. The 30 nmol dose resulted in a 1.6 log (96%) and 0.9 log (87%) reduction in viral titre when administered by the i.n. and s.c. routes, respectively compared to only a 0.5 log (65%) reduction when administered by the i.m. route, while the 10 nmol dose resulted in similar levels of viral clearance when administered by all routes (0.5–0.8 log).

The levels of split virus vaccine-induced viral clearance (Fig. 1B) were also route-dependent (p = 0.0004, by two-way ANOVA) and most effective when administered via the s.c. route. By this route, all doses tested (containing 0.03–10 µg HA) significantly reduced viral loads (p<0.001). In contrast, only doses above 0.1 µg of HA and 1.0 µg of HA administered by the i.m. and i.n. routes, respectively were able to significantly reduce viral titres. While significant levels of clearance were observed, none of the tested doses administered by any route was able to completely protect against infection indicating that all were sub-optimal.

**Figure 2:**
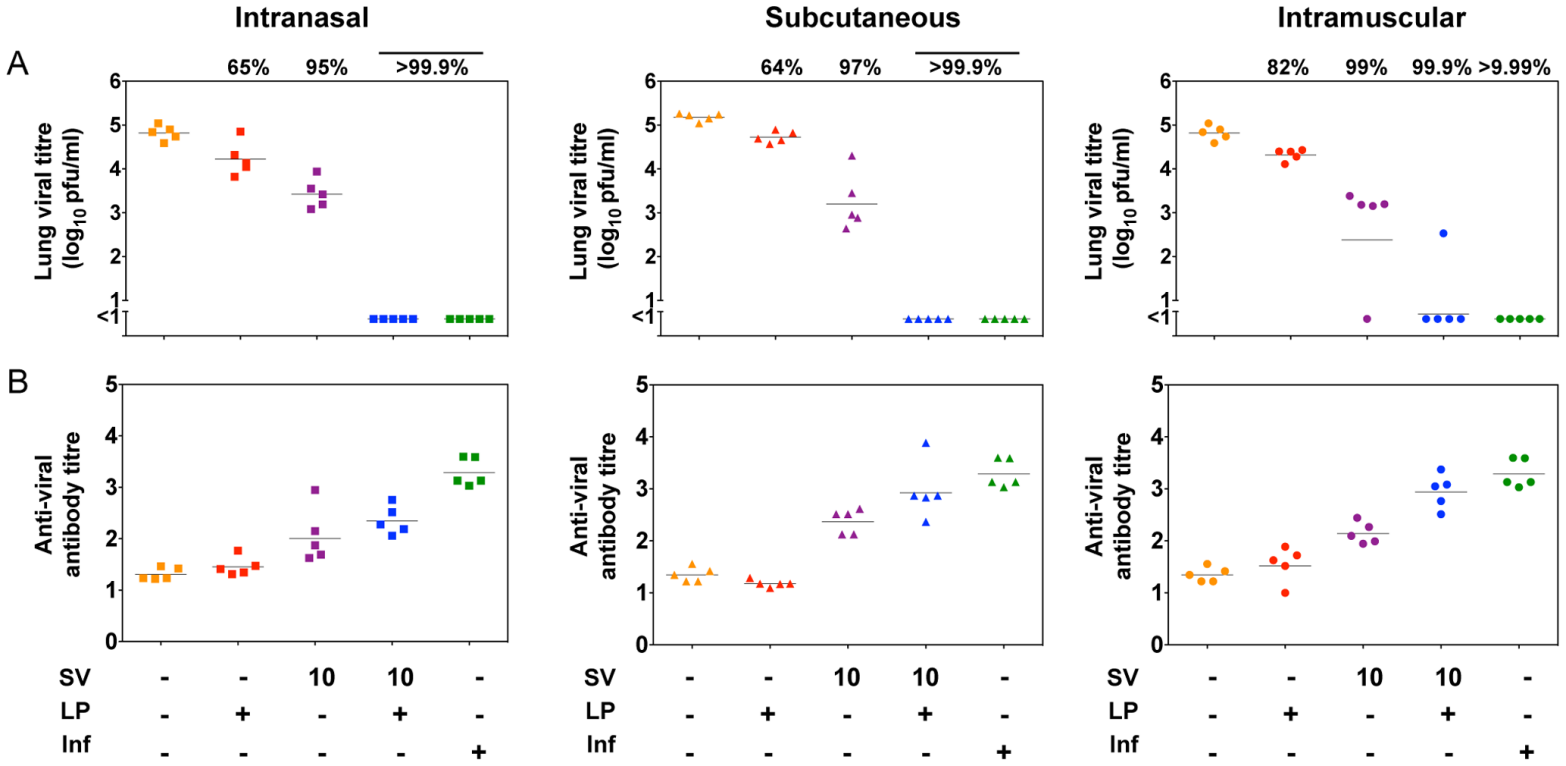
Viral clearance and antibody titre induced by immunisation with the high dose combination vaccine and its individual components. Mice were immunised once by the i.n. (squares), s.c. (triangles) or i.m. (circles) routes with 10 nmol lipopeptide (LP), 10 µg of HA as split virus (SV) or a combination of both components as indicated (+) or infected with 10^4.5^ pfu of virus (Inf). Control mice received PBS. One month post-immunisation, mice were challenged by the i.n. route with 10^4.5^ pfu of virus, lungs were removed 5 days later and pulmonary viral titre determined by plaque formation (A). Mice were bled prior to challenge, and the virus-specific antibody response in sera measured by ELISA (B). Symbols represent the titres for individual mice, and the line indicates the geometric mean for the group (n = 5). The numbers above the data sets refer to the average percentage decrease in viral load compared to PBS control mice.

**Figure 3:**
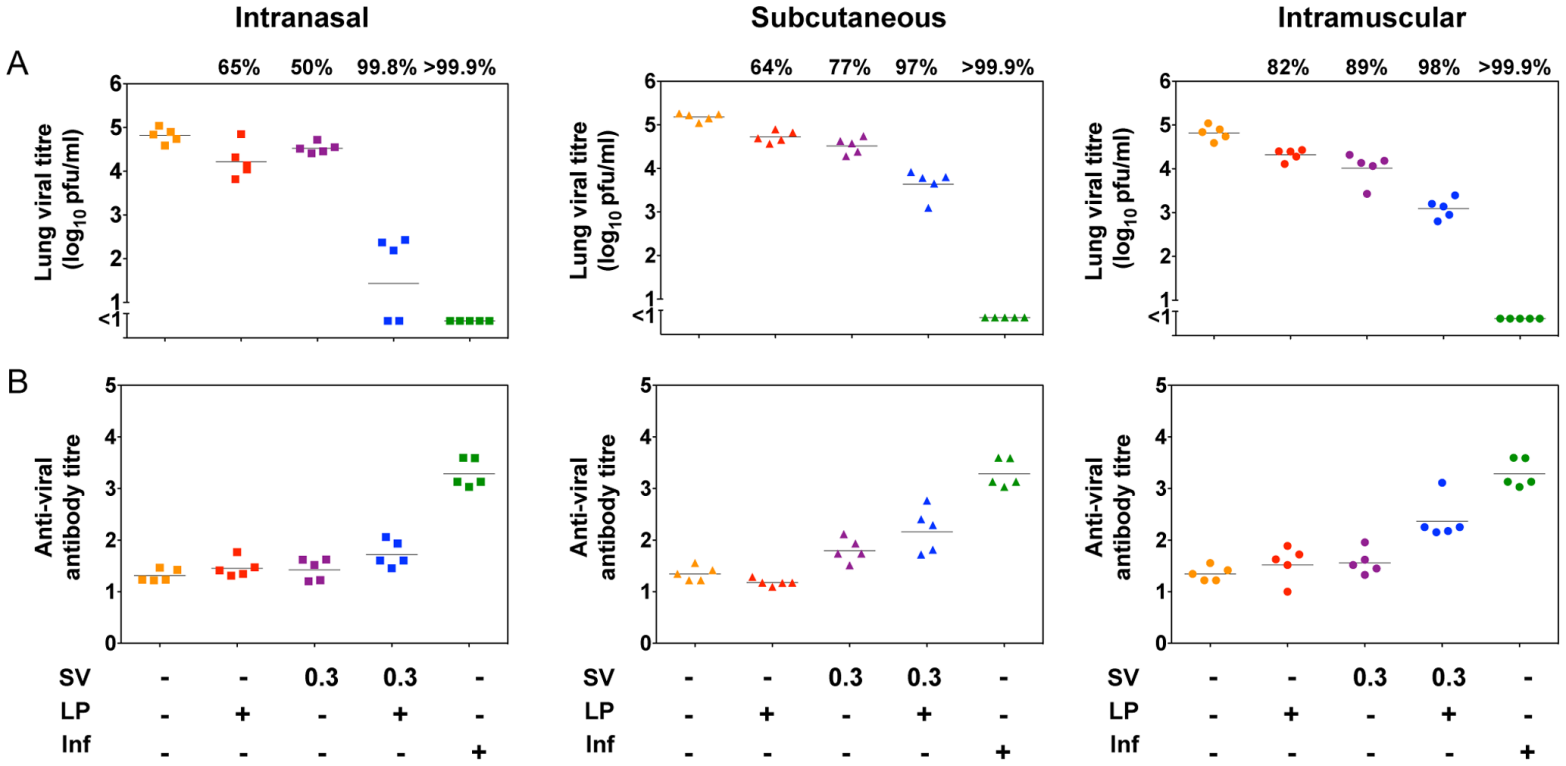
Viral clearance and antibody titre induced by immunisation with the low dose combination vaccine and its individual components. Mice were immunised once by the i.n. (squares), s.c. (triangles) or i.m. (circles) routes with 10 nmol lipopeptide (LP), 0.3 µg of HA as split virus (SV) or a combination of both components as indicated (+) or infected with 10^4.5^ pfu of virus (Inf). Control mice received PBS. One month post-immunisation, mice were challenged by the i.n. route with 10^4.5^ pfu of virus, lungs were removed 5 days later and pulmonary viral titre determined by plaque formation (A). Mice were bled prior to challenge, and the virus-specific antibody response in sera measured by ELISA (B). Symbols represent the titres for individual mice, and the line indicates the geometric mean for the group (n = 5). The numbers above the data sets refer to the average percentage decrease in viral load compared to PBS control mice.

These titrations were used to select particular doses of the lipopeptide and split virus to include in the combination vaccines. As all lipopeptide doses tested induced sub-optimal levels of protection, the 10 nmol dose was selected, as it was the lowest dose that significantly decreased the viral titres using each of the three routes. Two sub-optimal doses of split virus were selected for testing in the combination vaccines, a “high” dose of 10 µg of HA and “low” dose of 0.3 µg of HA. The split virus and the lipopeptides were mixed together just prior to administration and the resulting reduction in lung viral loads and the accompanying antibody responses were determined (Figs. 2 and 3).

### The high dose combination vaccination can reduce pulmonary viral loads through an increase in antibody titres

The levels of viral clearance observed with combination vaccines containing 10 nmol lipopeptide and 10 µg HA in split virus (referred to as the SV10-LP vaccine) were assessed in response to viral challenge one month post vaccination (Fig. 2A). By all three routes the SV10-LP combination vaccine resulted in reduced viral loads on day 5 post-challenge compared to either components alone (p<0.01). When administered by the i.n. or s.c. routes, the SV10-LP combination vaccine resulted in greater than 99.9% viral clearance, ie. no virus was detected, and only one of five mice immunised by the i.m. route was unable to completely clear the virus but this mouse had a greatly reduced pulmonary virus load.

Serum from the same mice taken one day prior to viral challenge (day 28) were assayed for virus-specific antibody by ELISA (Fig. 2B). Those mice immunised with the SV10-LP combination vaccine either by the s.c. or i.m. routes produced significantly higher antibody titres than mice immunised with the equivalent dose of split virus (SV10) alone (p<0.05) and to levels statistically similar to those induced by viral infection (p>0.05). A small increase in antibody titre was observed in mice immunised by the i.n. route yet levels were not significantly higher than the SV10 alone (p>0.05). As expected, when administered by any route the lipopeptide alone did not induce virus-specific antibodies.

### A reduction in viral load following the low dose combination vaccination can be observed in the absence of an increase in antibody levels

Similar assessment of the low dose combination vaccine containing 0.3 µg HA in split virus (SV0.3-LP) showed that administration by the i.n. route provided a 3.8 log (99.8%) reduction in viral titres, with two mice clearing virus to undetectable levels (Fig. 3A). Viral clearance observed with the combination vaccine delivered by either s.c. or i.m. routes, however, was not as extensive as by the i.n. route, though greater than the reduction seen with SV0.3 alone (p<0.001).

**Figure 4:**
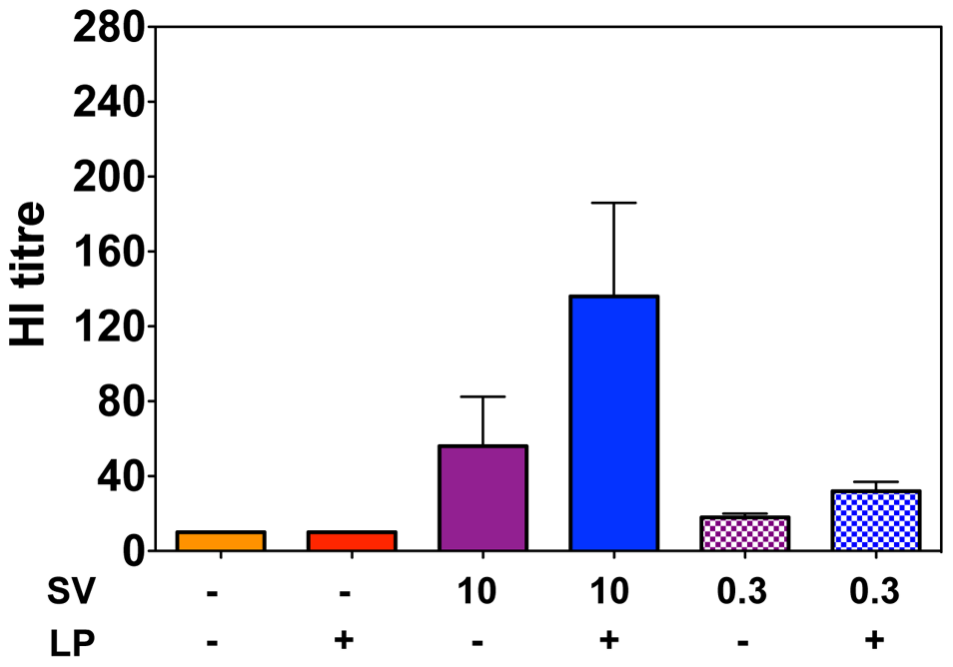
HI titer of serum from mice vaccinated by the i.n. route. Mice were immunized once with PBS, 10 nmol lipopeptide (LP), 10 or 0.3 µg of HA as split virus (SV) or combinations of these as indicated (+) by the i.n. route. Sera sampled one month later were tested for HI activity against the homologous virus. Bars represent the mean HI titers for groups of 5 mice and error bars indicate the standard error of the mean. Titers of <10, observed for all animals in the PBS and lipopeptide alone groups are designated as 10 for the purposes of statistical analysis.

Serum antibody titres determined at 28 days post-vaccination (Fig. 3B) revealed that the SV0.3 alone did not provide sufficient antigen stimulus to induce detectable virus-specific antibody within this timeframe by any of the delivery routes (p>0.05 versus PBS control). The addition of the lipopeptide to the lower dose of split virus slightly enhanced antibody titres when administered by the s.c. or i.m. routes (statistically significant only for i.m. p<0.01). Levels of antibody were not enhanced at all by the addition of the lipopeptide to SV0.3 when delivered by the i.n. route, indicating that the high level of viral clearance observed with the SV0.3-LP combination vaccine could not be attributed to antibody. Analysis of serum HI titers of mice inoculated with each of the different vaccines by the i.n. route (Fig. 4) parallels these data and shows the very low levels of functional antibody induced by the SV0.3 vaccines which is not significantly enhanced in the presence of the lipopeptide (p<0.05).

### Lipopeptide-specific CD8^+^ T cells can contribute to viral clearance in combination vaccines

**Figure 5:**
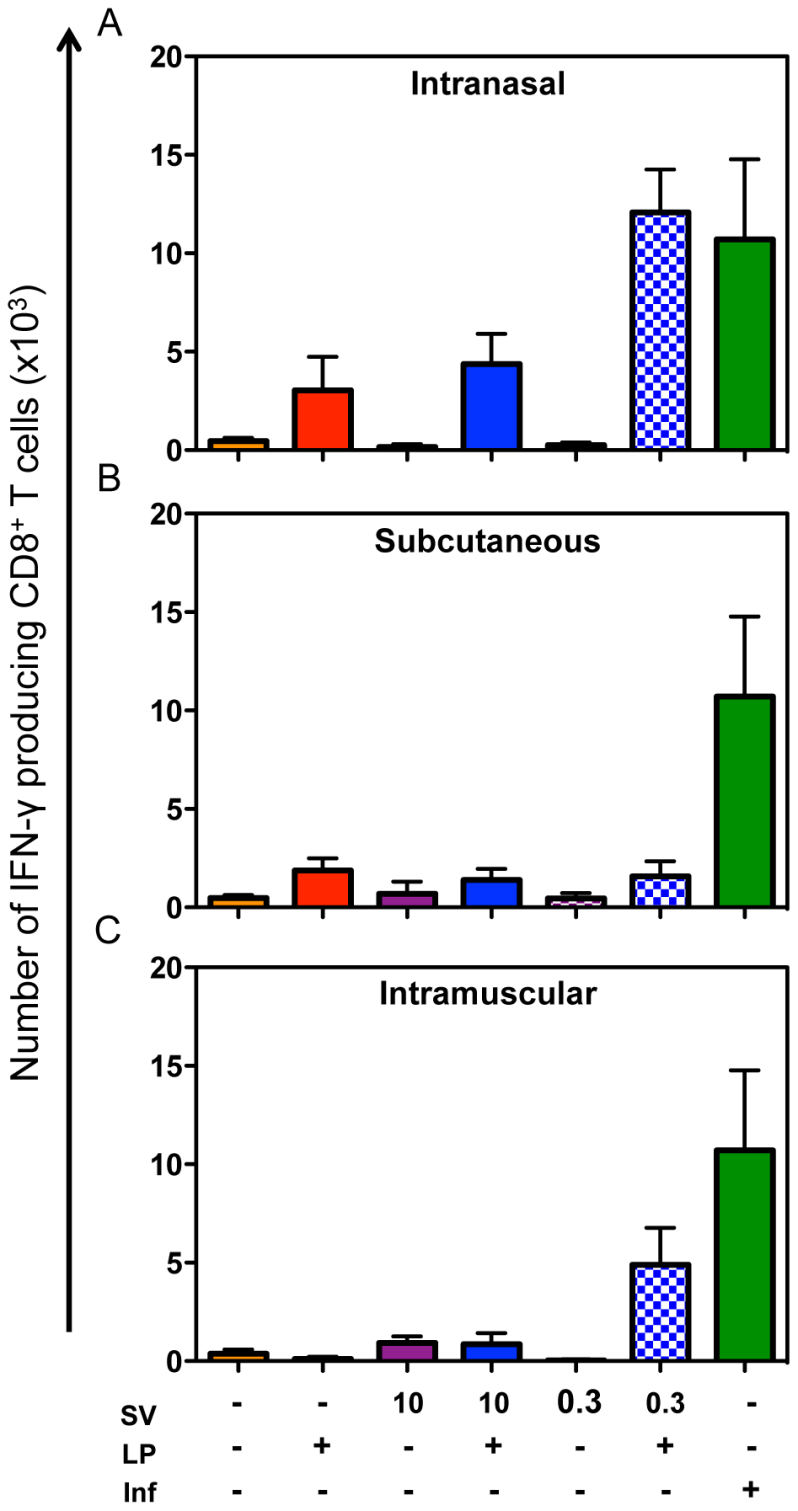
Influenza epitope-specific CD8^+^ T cells in the lungs of vaccinated mice after recall by viral challenge. Mice were immunised once by the i.n. (A), s.c. (B) or i.m. (C) routes with 10 nmol lipopeptide (LP), 10 or 0.3 µg of HA as split virus (SV) or combinations of these as indicated (+). Controls were infected with 10^4.5^ pfu of virus (Inf) or PBS. One month post immunisation, mice were challenged by the i.n. route with 10^4.5^ pfu of virus. The IFN-γ secreting CD8^+^ T cells in the lungs 5 days later that were specific for the TYQRTRALV epitope were enumerated by ICS. Data are presented as the total CD8^+^ T cells producing IFN-γ in response to peptide stimulation minus the number detected in the absence of stimulation. Bars represent the geometric mean for the group (n = 5) and error bars indicate the standard error.

To investigate whether CD8^+^ T cells were induced by the vaccines, the interferon γ-secreting CD8^+^ T cell populations, specific for the TYQRTRALV epitope in the lipopeptide, were assessed in the lungs of vaccinated mice 5 days after recall by the challenge virus (Fig. 5). In the PBS control, the pulmonary CD8^+^ T cell response to the challenge virus was only very weak on day 5 post-challenge (Fig. 5 A, B and C). In contrast, prior exposure to influenza infection induced a robust memory response that could be recalled within this time frame, as seen by a greatly enhanced level of CD8^+^ T cells specific for this epitope (which is immunodominant in the BALB/c response to infection) after challenge. As expected, the split virus vaccines alone did not induce any CD8^+^ T cell response, despite the presence of NP and thus the epitope, in these preparations.

Slightly higher levels of specific CD8^+^ T cells were detected in the lungs of mice vaccinated with the lipopeptide alone compared to PBS by the i.n. route (Fig. 5A) but not by the s.c. (Fig. 5B) or i.m. (Fig. 5C) routes. Neither the high or low dose combination vaccines induced significantly higher levels of specific CD8^+^ T cells than the PBS control when delivered by the s.c. route (p = 0.3). When delivered by the i.m. route, the SV0.3-LP vaccine (p<0.05) but not the SV10-LP vaccine, showed significant CD8^+^ T cell induction. The high dose combination vaccine delivered by the i.n. route induced a similar level of CD8^+^ T cells to the lipopeptide alone and, notably, the low dose combination gave a significantly higher level of CD8^+^ T cell induction (p<0.01 versus SV10-LP, <0.001 versus PBS), that was comparable to virus infection (p<0.05).

## Discussion

This study provides proof of principle for augmentation of viral clearing responses upon co-delivery of a T cell-inducing component with the split influenza virus vaccine. Enhancement of viral clearance was mediated through three separate mechanisms (i) boosting highly-specific antibodies titres to protective levels; (ii) inducing modest numbers of antibodies in combination with modest numbers of CD8^+^ T cells; or (iii) inducing robust cross-protective CD8^+^ T cell responses in the absence of antibodies.

In principle, if sufficient strain-specific neutralising antibody is present prior to virus exposure, infection would not become established. However, in practice this is rarely achieved by vaccination. Here, we modelled the situation where levels of vaccine-induced neutralising antibodies were sub-optimal for complete protection. Vaccines containing 10 µg (high dose) and 0.3 µg (low dose) HA in the form of split virus led to poor antibody production and incomplete viral clearance when they were delivered by each of the three routes tested. Addition of the T cell-inducing lipopeptide led to an improvement in viral clearance irrespective of dose or delivery route, although the degree of enhancement and the immune mediators involved differed. Table 1 summarises the levels of specific antibody and CD8^+^ T cells achieved with the combination vaccines, when delivered by different routes, and we consider below how these effectors might contribute to the observed viral clearance.

**Table 1:** Summary of immune responses induced by vaccination with combination vaccines.

Combination vaccine	Antibody levels	Lung CD8^+^ T cell levels	Viral clearance
SV10-LP i.n.	++^a^	++	+++
SV10-LP s.c.	+++	-	+++
SV10-LP i.m.	+++	-	+++
SV0.3-LP i.n.	-	+++	++
SV0.3-LP s.c.	+	-	+
SV0.3-LP i.m.	++	++	+
Infection	+++	+++	+++

a The responses to the combination vaccines have been scored on a 4-point scale from undetectable to statistically equivalent to viral infection: no response (-); weak response (+); moderate response (++); equivalent to viral infection (+++).

The addition of the lipopeptide to a “high” yet still sub-optimal dose of split virus significantly boosted antibody titres to levels comparable to that achieved by virus infection and conferred complete protection when administered by i.m. and s.c. routes. This is likely due to the adjuvant effect of the Pam2Cys component of the lipopeptide, which we have previously shown significantly enhances antibody responses to a variety of antigens [6], [12], [25], [30], [31] even to non-associated antigens [32]. Co-induction of lipopeptide-induced influenza-specific CD4^+^ T cells may also contribute to the increased antibody levels observed. In this regard, the combination vaccine could be used to minimise the dose of split virus required to achieve protection. This is highly significant when considering the cost benefit associated with fewer production cycles required for a given number of seasonal vaccine units and the flow-on decrease in time to vaccine rollout after production. Rapid vaccine production and the capacity for “dose sparing” is particularly important when immunisation of extremely large numbers of people such as in the event of a pandemic, where experience with H5N1 [33], pH1N109 [34] and recently H7N9 viruses [35], [36] leads us to predict lower-than-expected antigen yields and/or greatly reduced immunogenicity.

Even though these “high dose” combination vaccines induced high antibody responses when delivered by the i.m. and s.c. route, they did not induce CD8^+^ T cell responses in the lung, presumably because the virus was largely neutralised before infection was established, providing little stimulus for their induction. However, when the combination vaccine induced only moderate levels of antibody, which was the case for “high dose” vaccine delivered by the i.n. route, vaccine-induced memory CD8^+^ T cells were recalled in the lung by the challenge virus (Table 1). The complete viral clearance that resulted in this case highlights the fact that sub-neutralising levels of antibody and the lipopeptide-induced CD8^+^ T cells work synergistically to improve vaccine outcome.

The “low” dose combination vaccine showed only a very slight level of antibody enhancement above split virus alone when delivered by the s.c. route, and somewhat more by the I.M route. These antibody levels were much less than those induced by virus infection and were insufficient to completely clear the virus. Nevertheless, the viral load was reduced by up to 97%, which is at least a log more than the low dose split virus alone could achieve and may translate to significant reduction in disease duration and severity. Pulmonary CD8^+^ T cells were recalled by challenge in mice vaccinated with low dose combination vaccine delivered by the i.m. but not the s.c. route (Table 1). In contrast, by the i.n. route, there was no enhancement of the virtually negligible antibody response to the low dose split virus vaccine when combined with the lipopeptide, as measured by both ELISA and HI, and yet the viral load was decreased by almost three logs and virus was undetectable in 2 of the 5 mice. It is likely that the robust pulmonary CD8^+^ T cell response recalled by the challenge virus contributes to the clearance observed in this instance, although residual mucosal antibody responses if present may also play a role. Of note, the CD8+ T cell response was much greater than that recalled after vaccination with lipopeptide alone and as great as that recalled after virus infection itself. The ability of the low dose combination vaccine to induce CD8^+^ T cell numbers that were recalled to greater levels than that recalled after vaccination with lipopeptide alone again highlights the synergistic effects of the two arms of the immune response, with a possible role for co-induced split virus-primed CD4^+^ T cells in further expanding the pulmonary memory CD8^+^ T cell pool.

The accelerated clearance observed in mice immunised by the i.n. route compared to those immunised by the i.m. route suggests that the re-activation of the resident lung memory CD8^+^ T cell population may be faster acting than those that need to traffic from distal sites following priming by i.m. and s.c. routes, or are potentially of better quality. This suggests that the development of a lung memory population is advantageous in influenza vaccine design for conferring optimal levels of viral clearance. Due to the conserved nature of the T cell epitopes, such CD8^+^ T cell responses have the potential to be recalled by virtually all influenza A virus infections [37], [38] to allow clearance in the absence of antibody responses.

This study provides evidence that T cell and antibody responses are co-induced by novel combination vaccines and can act in concert to provide better control of influenza infection. Accessing the additional T cell arm of the immune response is especially important if seroconversion has not been adequate due to vaccine mismatch, waning B cell responsiveness, original antigenic sin, individuals not recently vaccinated and for emergence of a new subtype. The T cell-inducing Pam2Cys-based lipopeptide used here as an additive to the split virus vaccine contained only a single epitope for CD4^+^ and for CD8^+^ T cells. However, the technology has now advanced such that entire proteins can be adjuvanted with Pam2Cys [31], [32] to provide a source of multiple epitopes to account for MHC diversity within vaccinated populations. Our findings may pave the way for new generation influenza vaccines with the potential to greatly improve vaccine effectiveness against seasonal epidemics and to reduce the amount of split virus required. These combination vaccines may also provide the safety net required in the event of a pandemic strain entering the human population.
